# Reattempt Percutaneous Coronary Intervention of Chronic Total Occlusions after Prior Failures: A Single-Center Analysis of Strategies and Outcomes

**DOI:** 10.1155/2021/8835104

**Published:** 2021-04-20

**Authors:** Mingqiang Fu, Shufu Chang, Lei Ge, Dong Huang, Kang Yao, Feng Zhang, Qing Qin, Jianying Ma, Juying Qian, Junbo Ge

**Affiliations:** Department of Cardiology, Zhongshan Hospital, Fudan University, Shanghai Institute of Cardiovascular Diseases, Shanghai, China

## Abstract

**Objective:**

The initial recanalization rate of coronary chronic total occlusions (CTOs) is >85% when performed by experienced operators, but only 10% of prior failed CTO patients receive reattempted recanalization. This retrospective study analyzed the success rate and strategies used in reattempt percutaneous coronary intervention (PCI) of CTOs after prior failures.

**Methods:**

Overall, 206 patients with 212 CTOs were enrolled. All patients with prior recanalization failures received reattempt PCIs from January 2015 to March 2019 at Zhongshan Hospital, Fudan University. Data on clinical factors (age, sex, comorbidities, left ventricular ejection fraction, history of cigarette usage, and revascularization), angiographic characteristics of CTOs (target lesion, Japanese Chronic Total Occlusion (J-CTO) score, the morphology of CTO lesions, and collateral channel scale), strategies (procedural approach and use of devices), and major adverse events were obtained and analyzed.

**Results:**

The mean age of enrolled patients was 60.96 ± 12.36 years, with a male predominance of 90.3%. Of the patients, 47.1% had a prior myocardial infarction and 70.4% underwent stent implantation previously, while the in-stent occlusion rate was 6.6%. CTOs were primarily localized in the left anterior descending artery (43.9%) and the right coronary artery (43.9%). 80.7% of lesions were classified as very difficult (J-CTO score ≥3), and the overall success rate was 81.1%. In multivariable regression analysis, J-CTO score, collateral channel scale, application of coronary multispiral computed tomography angiography, dual injection, intravascular ultrasound, active greeting technique, parallel wiring, and CTO morphology were predictors of recanalization success. There were no significant differences in rates of procedural complications between the final recanalization success and failure groups.

**Conclusions:**

Recanalization of complex CTOs is associated with high success rate and low complication rates when performed by high-volume CTO operators and after multiple reattempts.

## 1. Introduction

Chronic total occlusion (CTO) lesions are identified in 18%–33% of all patients referred for coronary angiography [1, 2]. Also, the presence of concurrent CTO is a strong predictor for both short-term and long-term mortality [3, 4]. In the era of interventional therapy, the indications for CTO revascularization are similar to those for severe stenosis according to European Society of Cardiology guidelines [5]. Compared with optimal medical therapy alone, the combination of CTO revascularization with optimal medical therapy is associated with significant ischemia relief, left ventricular function improvement, and a better quality of life [6–8]. In recent years, the development of contemporary techniques and devices has substantially improved the initial success rate >85% for CTO-PCI in unselected clinically indicated cases with ≈3% risk for major in-hospital complications when performed by highly experienced operators [9–11]. However, outcomes are less favorable at less-experienced centers with an initial success rate of around 60% [12, 13]; the procedural failed CTO patients either receive medical therapy still suffering symptomatic ischemia or are advised to undergo reattempt PCI or more traumatic coronary artery bypass grafting (CABG) surgery [14].

It is unclear whether reattempted PCI for CTO lesions is efficacious and safe by expert operators because the prior failure of percutaneous revascularization of CTO has been identified as an independent predictor for failure at subsequent attempts [15]. To our knowledge, there have been scattered reports focusing on strategies and outcomes for reattempted CTO-PCIs following previously failed procedures. In the present study, we sought to define outcomes and predictors of reattempted CTO-PCI success performed by highly skilled operators at our center.

## 2. Methods

### 2.1. Study Population and Data Collection

Between January 1, 2015, and March 31, 2019, a total of 206 consecutive patients with CTO lesions after prior PCI failures at local hospitals or our center with a history of multiple recanalization attempts were included. Patient data on baseline clinical characteristics, coronary angiographic information, procedural strategies, and complications were obtained.

### 2.2. Study Definitions and Evaluation of Strategies and Outcomes

Coronary chronic total occlusions (CTOs) are defined as 100% occlusions with Thrombolysis in Myocardial Infarction (TIMI) 0 flow with at least a 3-month duration [2, 16]. Occlusion duration was estimated based on the first onset of ischemic symptoms, prior history of myocardial infarction (MI) in the target vessel territory, comparison with a prior angiogram, or as the presence of bridging collateral vessels.

Lesion calcification was classified as mild (spots), moderate (≤50% of the reference lesion diameter), or severe (>50% of the reference lesion diameter). Moderate proximal vessel tortuosity was defined as the presence of at least 2 bends >70° or 1 bend >90°, and severe tortuosity was defined as 2 bends >90° or 1 bend >120° in the CTO vessel. Blunt or no stump was defined as the absence of tapering or a funnel shape at the proximal or distal cap.

Collaterals included septal, epicardial, ipsilateral, and saphenous vein graft. Angiographic assessment of a collateral connection (CC) was based on Werner's classification: CC0, no continuous connection between the donor and recipient artery; CC1, continuous, thread-like connection; and CC2, continuous small side branch-like size of the collateral throughout its course [17].

The J-CTO (Multicenter CTO Registry in Japan) score was determined by assigning 1 point to each of the following factors: blunt entry stump, calcification, bend >45^o^, occlusion length ≥20 mm, and previous failed attempt. The total number of points was added to stratify lesions into 4 difficulty groups: easy (J-CTO score of 0), intermediate (J-CTO score of 1), difficult (J-CTO score of 2), and very difficult (J-CTO score ≥3) [18].

Recanalization strategies of CTO include two paths (anterograde or retrograde) and two ways: through the true lumen or the subintimal space (with dissection and reentry to the true lumen). The hybrid strategy involved using both antegrade and retrograde approaches or switching from the originally selected approach to the other approach [19, 20]. The operators determined which strategy to use.

Technical success was defined as the ability to cross an occluded segment and successfully open the artery (restoration of TIMI flow grade of 2 or 3) with residual stenosis of <30%. Procedural success was defined as the achievement of technical success without any in-hospital major adverse cardiac events (MACE) [19].

Procedural complications included coronary artery dissection, hematoma and/or perforation, and tamponade requiring either pericardiocentesis or surgery. Contrast-induced nephropathy (CIN) was defined as an absolute increase of ≥0.5 mg/dL or a relative increase of at least 25% in serum creatinine levels within 48 to 72 h of intravenous administration of an iodinated contrast agent in the absence of other identifiable causes [21]. In-hospital MACE included any of the following before discharge: death, MI (defined using the fourth universal definition of type 4a), and recurrent symptoms requiring immediate repeat target vessel revascularization with PCI or CABG.

### 2.3. Statistical Analysis

Categorical variables are presented as numbers (percentages) and compared using Pearson chi-square or Fisher exact tests. Continuous variables are presented as mean ± standard deviation if normally distributed or median with an interquartile range if nonnormally distributed and compared using Student's *t* or Mann–Whitney U test, as appropriate. All indices with a *p* value <0.1 in the univariate analysis were included in a multivariable logistic regression analysis to test reattempted CTO-PCI success with baseline clinical and angiographic characteristics as well as strategies. All data were analyzed using SPSS 20.0 (IBM SPSS Inc., Armonk, NY). A two-sided *p* value of 0.05 was considered statistically significant.

## 3. Results

### 3.1. Clinical Characteristics

A total of 206 patients were enrolled in the study. Nearly 90% of the patients for reattempts (178 out of 206 patients) were referred from other centers or other operators. Reattempts of CTOs were required to achieve recanalization in 166 patients, while recanalization could not be achieved in 40 patients even after multiple reattempts. The mean age of patients was 60.96 ± 12.36 years, with a male predominance of 90.3%. Regarding comorbidities, 69.4% had hypertension, 33.5% had diabetes mellitus, 20.9% had hyperlipidemia, 54.9% were smokers, and 47.1% had prior MI. Notably, 70.4% of patients had previously received stent implantation, and 5.8% had received CABG. There were no significant differences in the distribution of clinical characteristics between patients with final successful reattempts and final failed reattempts (Table 1).

### 3.2. Angiographic Characteristics

There were 212 initially failed CTOs in 206 patients, of which 172 CTOs in 166 patients were successfully recanalized after reattempts, whereas recanalization of 40 CTOs in 40 patients finally failed. Typically, target CTO lesions were primarily located in the left anterior descending coronary artery (43.9%) or right coronary artery (43.9%), and 6.6% were in-stent occlusions. Of the CTOs, 54.2% scaled CC grade 1 and 43.9% scaled CC grade 2, and more than two-thirds were multiple coronary vessel disease. Among the included CTOs, 80.7% were technically very difficult (J-CTO score ≥3, prior failure as part of the score derivation). By detailed evaluation of each characteristic of J-CTO score, 75.5% were occlusion lesions >20 mm, 27.8% were tortuous, 47.2% were blunt proximal cap, 24.1% were blunt distal cap, and 9.4% were severely calcified.

Compared with final successful procedures, final failed lesions had considerably higher J-CTO score (4.00 ± 0.72 vs. 3.33 ± 0.87, *p* < 0.001), less-useful CCs (CC grade 0 : 7.5% vs. 0.6%, *p* = 0.004; CC grade 2 : 27.5% vs. 47.7%, *p* = 0.021), and higher triple vessel disease rate (67.5% vs. 23.5%, *p*<0.001), which indicated the final failed lesions had more complicated morphology (Table 2).

### 3.3. Strategies between Successful and Failed Cases

**Figure 1:**
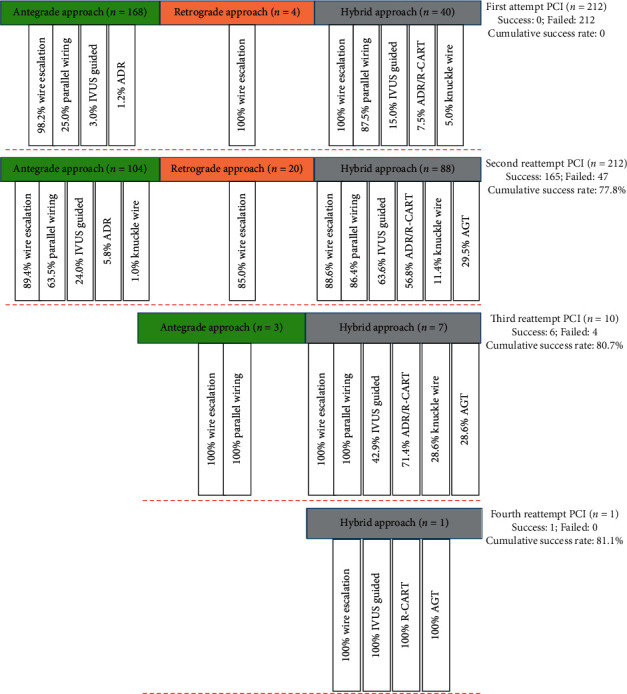
Distributions of strategies and crossing techniques. PCI, percutaneous coronary intervention; IVUS, intravascular ultrasound; ADR, antegrade dissection and reentry (CrossBoss and/or Stingray device); R-CART, reverse-controlled antegrade or retrograde subintimal tracking; and AGT, active greeting technique.

Of all reattempted CTO procedures, second-attempt recanalization was achieved in 165 lesions, third-attempt recanalization was acquired in 6 lesions, and fourth-attempt recanalization was successful in 1 lesion, and the overall success rate was 81.1% (172/212 lesions; Figure 1).

At initial attempts, antegrade wiring was required in 79.2% (168/212) of patients, with a dual injection rate of 18.4% (39/212). When reattempts were implemented, antegrade wiring was required in 48.6% (103/212) of patients, with a dual injection rate of 67.9% (144/212). The most used collateral in retrograde wiring was the septal connection (42.0%). 10.8% (23/212) of reattempted cases involved tip injection, 38.7% (82/212) cases required intravascular ultrasound (IVUS) to facilitate CTO wiring, and 16.0% (34/212) cases required reverse-controlled antegrade or retrograde subintimal tracking (R-CART). CrossBoss and/or Stringray devices (Boston Scientific Corporation) were used in 13 cases (6.1%), and active greeting technique (AGT) was used in 26 cases (12.3%).

The intervals for a second recanalization attempt between finally successful and failed cases were comparable (4.08 ± 5.26 months vs. 3.85 ± 5.34 months, *p* = 0.799). Compared with final failed cases, final successful cases involved greater apply of coronary multispiral computed tomography angiography (CCTA, 27.1% vs. 7.5%, *p* < 0.001) and dual injection (73.8% vs. 42.5%, *p* = 0.003) to acquire comprehensive anatomical information of target lesions. Meanwhile, implementation of IVUS, R-CART, and AGT was more common in final successful cases. There were no significant differences in comparison to the procedural approach, collaterals used, rotational atherectomy and anterograde dissection and reentry (ADR; use of CrossBoss and/or Stringray CTO reentry devices) maneuvers (Table 3).

### 3.4. Safety and Complications

As is presented in Table 4, reattempts were accompanied by greater contrast and X-ray doses than the initial attempts. Compared to finally recanalized lesions, final failed cases required more contrast (317.38 ± 184.42 ml vs. 297.38 ± 148.96 ml, *p* < 0.05) and increased dose of X-ray (2491.28 ± 1178.14 mGy vs. 2319.11 ± 1397.31 mGy, *p* < 0.05) and were time consuming (48.82 ± 33.66 min. vs. 46.89 ± 34.10 min., *p* < 0.05).

7.5% of patients in the final failed group experienced tamponade during the procedure, while other procedure-related complications and MACE were comparable between final successful and failed cases. Notably, the contrast-induced nephropathy (CIN) rate was considerably higher in the final successful group patients than in the final failed group patients (Table 5).

### 3.5. Reasons for Initial Procedural Failure and Predictors of Success

**Figure 2:**
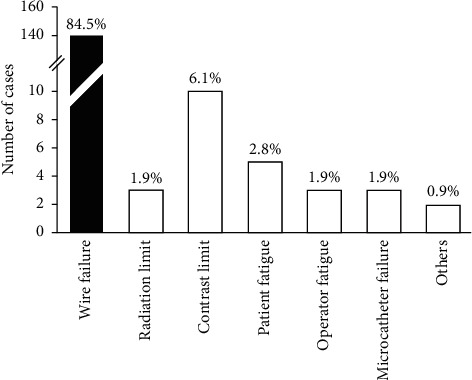
Distribution of procedural failures.

The reasons for initial procedural failures are summarized in Figure 2; wire failure was the predominant reason to terminate the procedure (84.5%), followed by a contrast limit (6.1%). Rare reasons included radiation limit, operator or patient fatigue, and microcatheter failure.

To determine which indexes were mainly affecting procedural outcomes, we conducted correlation and logistic regression analyses. Multivariable regression analysis revealed that J-CTO score, CC scale, CCTA, dual injection, IVUS, AGT, parallel wiring, and CTO morphology were predictors of recanalization success. In contrast, sex, age, target lesion location, time of interval, procedural approach, ADR/RDR, rotational atherectomy, bridging collaterals, in-stent occlusion, CTO bend, comorbidities, smoke, LVEF, renal function, and previous histories of MI and revascularization appeared less important (Table 6). Correlation analysis revealed that higher J-CTO score, collaterals existed in proximal or distal lesions, lesion length ≥20 mm, and severe lesion calcification were negatively correlated with procedural success. In contrast, higher CC scale, preoperation CCTA application, dual injection, IVUS-guided wiring, AGT, and tapered cap existed in both proximal and distal lesions were positively correlated with successful recanalization (supplementary table).

## 4. Discussion

The main findings of the present study were that prior failed CTO lesions were associated with higher complexity of morphology; however, 81.1% of CTOs could be recanalized safely and effectively by experienced operators at repeat attempts. There was a definite relationship between lesion complexity and the increasing need for multiple approaches and technologies during CTO-PCI to achieve success.

Multicenter CTO Registry in Japan proved that “retry” was an unsuccessful predictor for initial failed CTOs [15]; on the other hand, CTO patients were less likely to choose for re-PCI potentially due to low expectations of procedural success and concerns regarding complications; thus, merely 10% patients were referred for PCI other than optimal medical therapy or CABG [22]. A meta-analysis of 25 studies compared successful (71%) with failed (29%) CTO-PCIs in 28486 patients. During a mean follow-up of 3.11 years, successful CTO-PCI was associated with lower mortality, less residual angina, lower risk for stroke, and less need for subsequent CABG with comparison to failed procedures [23]. According to related guidelines and from our experience, successful opening of CTOs, even repeated attempts, would greatly benefit patients with fewer complications.

Generally, initial failure of recanalization for CTOs indicates that lesion morphologies are more unfavorable for wire or microcatheter crossing, which required prolonged procedural time, increased probability of complications, and reduced reimbursement. All of these factors rendered reattempt recanalization of CTO lesions considerably difficult and failed. As was demonstrated in our analysis and the study by Tanabe et al., the inability of guidewire passage through CTO lesions was the most common reason for the failure of re-PCI of CTOs [24]. The main reason of wire failure lied in lesion anatomy, as is shown in Table 2; >50% of lesions had an ambiguous stump, two-thirds of CTOs had an occlusion length >20 mm, 56.1% of patients had unavailable collaterals, and 37.8% had triple vessel disease or left main plus multivessel disease.

Given increased complexity and risk, the retrograde approach was usually performed when antegrade crossing attempts fail or carried more risk. In the antegrade approach era, the low success rate of 65%–70% (even lower rate for reattempts) and high complication rate were the main barriers to CTO-PCIs [25]. The advent of the retrograde approach circumvented the limitations of the antegrade approach. Moreover, increasing knowledge and expertise could improve complex CTO-PCIs' success rate when the conventional antegrade approach was deemed unsafe or inefficient [1, 26]. However, the choice between antegrade and retrograde approaches using wire escalation or dissection reentry methods depended on the CTO anatomy and the operator's experience, favoring timely use of a hybrid algorithm if the procedure did not progress smoothly [27]. It was very important to determine the strategy for a reattempt CTO-PCI with reference to detailed lesion information by preprocedural CCTA and dual injection during the operation. In this study, preprocedural CCTA occupied 27.1%, and dual injection was employed in 73.8% of patients in final successful reattempts to provide detailed lesion characteristics, including lesion length, proximal and distal cap morphology, and the extension and morphology of the collateral branches, which helped drive the strategy choice or switch. CTO crossing is often easier in the retrograde direction because the distal CTO cap is often less fibrocalcific, more tapered, and exposed to lower pressure than the proximal cap [28].

In addition to choosing an appropriate procedural approach and strategy, it is noteworthy that a suitable combination of techniques and devices was also of high importance. In our analysis of final successful reattempts, IVUS-guided CTO wiring to resolve proximal cap ambiguity, R-CART, AGT, and CrossBoss and Stingray systems were used at a high percentage. Particularly, retrograde wire externalization facilitated by AGT appeared feasible and safe at our center [29].

The optimal time threshold for change continued to be debated and heavily dependent on operator expertise. The operators decided the use of all available crossing strategies and prompt changes in techniques and equipment. Expertise in all CTO crossing strategies was crucial for achieving the best possible procedural outcomes, especially for complex lesions. Antegrade wire escalation was often associated with lower complication rates and was often preferred as the initial crossing strategy if technically feasible. However, for complicated CTO lesions, antegrade wiring was related to a low success rate. Retrograde and antegrade dissection and reentry remained essential for the successful recanalization of more complex CTOs. In subsequent reattempts, all operators who had performed a career minimum of 300 CTO cases mastered all the available techniques and the appropriate combination of devices (antegrade dissection reentry (ADR) and retrograde dissection reentry (RDR)) in the hybrid algorithm. Referral to a more experienced operator could be helpful, especially if the CTO is of high complexity or there was a previous failed attempt.

The overall incidences of procedure-related complications and MACE were low in this study, similar to previous reports [24, 30], except that the CIN rate was higher in both finally successful and failed reattempted patients. Consequently, CTO-PCI in reattempted lesions was as safe as initially attempted CTO-PCI.

It should be noted that forty CTO lesions in forty patients remained unopened despite multiple attempts, twenty-four patients were referred to CABG, and the rest chose medical therapy with residual symptoms. These lesions were compromised with high J-CTO scores and lacked fair interventional collaterals, which means no chance for an antegrade or retrograde approach, and successful interventional revascularization might be limited even though expert operators endeavored reattempted CTO-PCIs.

### 4.1. Study Limitations

Although we aimed to take an all-comer population, most operators were reliant upon referrals from other cardiologists, and the treatment policy was largely determined by the individual operator. Therefore, these results would be unlikely generalizable to other less-experienced CTO-PCI operators.

## 5. Conclusions

Considering lesion complexity and prior failure, reattempted CTO-PCIs could still achieve an overall success rate of 81.1% by high-volume operators with acceptable complications.

## Figures and Tables

**Table 1:** Baseline clinical characteristics of the patients.

Category	Overall (*n* = 206)	Final success (*n* = 166)	Final failure (*n* = 40)	*p* value
Age (years)	60.96 ± 12.36	61.04 ± 12.34	60.62 ± 12.60	0.851
Male (%)	186 (90.3)	150 (90.4)	36 (90.0)	0.945
HTN (%)	143 (69.4)	111 (66.9)	32 (80.0)	0.106
DM (%)	69 (33.5)	58 (34.9)	11 (27.5)	0.371
HL (%)	43 (20.9)	35 (21.1)	8 (20.0)	0.880
Smokers (%)	113 (54.9)	93 (56.0)	20 (50.0)	0.492
LVEF (%)	56.37 ± 9.90	55.99 ± 9.71	57.97 ± 10.61	0.255
≤40 (%)	17 (8.3)	13 (7.8)	4 (10.0)	0.655
>40 (%)	189 (91.7)	153 (92.2)	36 (90.0)	0.655
eGFR (mL/min/1.73 m^2^)	82.71 ± 22.12	83.25 ± 21.49	80.45 ± 24.73	0.473
≤60 (%)	36 (17.5)	28 (16.9)	8 (20.0)	0.640
>60 (%)	170 (82.5)	138 (83.1)	32 (80.0)	0.640
Prior MI (%)	97 (47.1)	74 (44.6)	23 (57.5)	0.142
Prior PCI (%)	145 (70.4)	120 (72.3)	25 (62.5)	0.223
Prior CABG (%)	12 (5.8)	12 (7.2)	0 (0.0)	0.169

Values are mean ± SD or percentages. *p* value stands for comparison between final successful and final failed patients' clinical characteristics. HTN, hypertension; DM, diabetes mellitus; HL, hyperlipidemia (low-density lipoprotein cholesterol ≥3.4 mmol/L); LVEF, left ventricular ejection fraction; eGFR, estimated glomerular filtration rate; MI, myocardial infarction; PCI, percutaneous coronary intervention; CABG, coronary artery bypass grafting.

**Table 2:** Baseline angiographic characteristics of CTO lesions.

Category	Overall (*n* = 212)	Final success (*n* = 172)	Final failure (*n* = 40)	*p* value
CTO target vessels				
LM (%)	1 (0.4)	1 (0.6)	0 (0.0)	0.629
LAD (%)	93 (43.9)	79 (45.9)	14 (35.0)	0.210
LCX (%)	25 (11.8)	20 (11.6)	5 (12.5)	0.878
RCA (%)	93 (43.9)	72 (41.9)	21 (52.5)	0.222
In-stent occlusion (%)	14 (6.6)	10 (5.8)	4 (10.0)	0.337

Collateral channels				
CC 0 (%)	4 (1.9)	1 (0.6)	3 (7.5)	0.004
CC 1 (%)	115 (54.2)	89 (51.7)	26 (65.0)	0.130
CC 2 (%)	93 (43.9)	82 (47.7)	11 (27.5)	0.021
J-CTO score	3.45 ± 0.88	3.33 ± 0.87	4.00 ± 0.72	<0.001
<3 (%)	41 (19.3)	33 (19.2)	8 (20.0)	0.907
≥3 (%)	171 (80.7)	139 (80.8)	32 (80.0)	0.907

Number of diseased vessels				
Single VD	44 (21.4)	41 (24.7)	3 (7.5)	0.022
Double VD	84 (40.8)	75 (45.2)	9 (22.5)	0.014
Triple VD	66 (32.0)	39 (23.5)	27 (67.5)	<0.001
LM + multiple VD	12 (5.8)	11 (6.6)	1 (2.5)	0.471

Morphology of the proximal cap				
Blunt	100 (47.2)	70 (40.7)	30 (75.0)	<0.001
Side branch at the proximal cap	153 (72.2)	118 (68.6)	35 (87.5)	0.016

Target lesion morphology				
Tortuosity of the CTO lesion	59 (27.8)	47 (27.3)	12 (30.0)	0.734
CTO length ≥20 mm	160 (75.5)	122 (70.9)	38 (95.0)	0.001

Lesion calcification				
Mild	169 (79.7)	141 (81.9)	28 (70.0)	0.090
Moderate	23 (10.9)	19 (11.1)	4 (10.0)	0.848
Severe	20 (9.4)	12 (7.0)	8 (20.0)	0.025

Morphology of the distal cap				
Blunt	51 (24.1)	24 (14.0)	27 (67.5)	<0.001
Side branch at the distal cap	83 (39.2)	59 (34.3)	24 (60.0)	0.003

Values are mean ± SD or percentage. *p* value stands for comparison between final successful and final failed angiographic characteristics of CTO lesions. LM, left main artery; LAD, left anterior descending coronary artery; LCX, left circumflex coronary artery; RCA, right coronary artery; CC, collateral connection; VD, vessel disease.

**Table 3:** Strategies and differences between final successful and failed cases.

	Final successful reattempts (*n* = 172)	Final failed reattempts (*n* = 40)	*p* value
Month of intervals	4.08 ± 5.26	3.85 ± 5.34	0.799
Technical success	172 (100)	0 (0.0)	–
Procedural success	169 (98.3)	0 (0.0)	–
CCTA preprocedure	45 (27.1)	3 (7.5)	<0.001
Dual injection	127 (73.8)	17 (42.5)	0.003

Procedural approach			
Antegrade approach	88 (51.2)	15 (37.5)	0.119
Retrograde approach	16 (9.3)	4 (10.0)	0.892
Hybrid approach	68 (39.5)	21 (52.5)	0.135

Collaterals used in retrograde wiring			
Septal	69 (40.1)	20 (50.0)	0.254
Epicardial	16 (9.3)	7 (17.5)	0.157
Bypass graft/ipsilateral	3 (1.7)	0 (0.0)	0.402

Tip injection	20 (11.6)	3 (7.5)	0.580
IVUS-guided wiring	74 (43.0)	8 (20.0)	0.007
R-CART	32 (18.6)	2 (5.0)	0.035
AGT	26 (15.1)	0 (0.0)	0.018
Rotational atherectomy	9 (5.2)	0 (0.0)	0.213
CrossBoss or Stingray	11 (6.4)	2 (5.0)	0.740

Values are mean ± SD or percentage. CCTA, coronary multispiral computed tomography angiography; IVUS, intravascular ultrasound; R-CART, reverse-controlled antegrade or retrograde subintimal tracking; AGT, active greeting technique.

**Table 4:** Contrast and radiation differences.

	Final successful cases (*n* = 172)		Final failed cases (*n* = 40)	
	Initial attempt	Successful reattempt	Initial attempt	Failed reattempt
Contrast (mL)	249.37 ± 142.76	297.38 ± 148.96^a^	229.12 ± 162.61	317.38 ± 184.42^a,b^
Dose of X-ray (mGy)	1739.69 ± 1110.72	2319.11 ± 1397.31^a^	1622.36 ± 988.02	2491.28 ± 1178.14^a,b^
Time of X-ray (min)	38.63 ± 23.59	46.89 ± 34.10^a^	36.88 ± 20.32	48.82 ± 33.66^a,b^

Values are mean ± SD. mL, milliliter; mGy, milligray. a, compared to the index attempt, *p* < 0.01; b, comparison of failed reattempt vs. successful reattempt, *p* < 0.05.

**Table 5:** Complications between final successful and failed reattempted cases.

	Final successful reattempts (*n* = 172)	Final failed reattempts (*n* = 40)	*p* value
Procedural complications	25 (14.5)	8 (20.0)	0.390
Tamponade	0	3 (7.5)	0.004
Dissection/hematoma/perforation	23 (13.4)	5 (12.5)	0.883
Others (puncture site complication, wire broken, and side branch occlusion)	2 (1.2)	0	0.493
CIN	16 (9.6)	2 (5)	<0.001
MACE	3	1	0.570

Values are mean ± SD or percentage. CIN, contrast-induced nephropathy; MACE, major adverse cardiac events (including death, myocardial infarction, and recurrent symptoms requiring immediate repeat target vessel revascularization).

**Table 6:** Multivariable logistic regression analysis.

Parameter	*p* value
Sex	0.807
Age	0.983
Target lesion	0.206
J-CTO score	0.009
Month of interval	0.076
CC scale	0.019
CCTA	0.013
Dual injection	0.002
Procedural approach	0.117
IVUS-guided wiring	0.018
ADR/RDR	0.070
AGT	0.012
Parallel wiring	0.023
Rotational atherectomy	0.229
Bridging collaterals usage	0.127
In-stent occlusion	0.342
Proximal cap morphology	<0.001
Proximal collateral	0.018
CTO length	0.001
CTO bend	0.693
Distal cap morphology	<0.001
Distal collateral	0.003
Calcification	0.012
HTN	0.066
DM	0.477
HG	0.820
Smokers	0.482
LVEF	0.521
eGFR	0.583
Prior MI	0.119
Prior CABG	0.084
Prior PCI	0.211

CC, collateral connection; CCTA, coronary multispiral computed tomography angiography; IVUS, intravascular ultrasound; ADR, anterograde dissection and reentry; RDR, retrograde dissection and reentry; AGT, active greeting technique; HTN, hypertension; DM, diabetes mellitus; HG, hyperlipidemia; LVEF, left ventricular ejection fraction; eGFR, estimated glomerular filtration rate; MI, myocardial infarction; CABG, coronary artery bypass grafting; PCI, percutaneous coronary intervention.

## Data Availability

The primary data are available on request from the author.

## Supplementary Materials

Supplementary Materials Correlation analysis is conducted to determine the related indexes with procedural outcomes. It revealed that higher J-CTO score, collaterals existed in proximal or distal lesions, lesion length ≥20 mm, and severe lesion calcification were negatively correlated with procedural success. In contrast, higher CC scale, preoperation CCTA application, dual injection, IVUS-guided wiring, AGT, and tapered cap existed in both proximal and distal lesions were positively correlated with successful recanalization.
