# Supplementary material for: Reattempt Percutaneous Coronary Intervention of Chronic Total Occlusions after Prior Failures: A Single-Center Analysis of Strategies and Outcomes
Source: J Interv Cardiol. 2021 Apr 20;2021:8835104. doi: 10.1155/2021/8835104 (PMC8079192; doi:10.1155/2021/8835104)
Supplement: Supplementary Materials — Correlation analysis is conducted to determine the related indexes with procedural outcomes. It revealed that higher J-CTO score, collaterals existed in proximal or distal lesions, lesion length ≥20 mm, and severe lesion calcification were negatively correlated with procedural success. In contrast, higher CC scale, preoperation CCTA application, dual injection, IVUS-guided wiring, AGT, and tapered cap existed in both proximal and distal lesions were positively correlated with successful recanalization. [file 8835104.f1.docx]

Correlation analysis

| Parameter | correlation coefficient | p value |
| --- | --- | --- |
| Sex | 0.009 | 0.892 |
| Age | 0.008 | 0.903 |
| Target lesion | -0.085 | 0.218 |
| J-CTO score | -0.178 | 0.009 |
| Month of interval | -0.125 | 0.070 |
| CC scale | 0.159 | 0.020 |
| CCTA | 0.174 | 0.011 |
| Dual injection | 0.207 | 0.002 |
| Procedural approach | -0.108 | 0.118 |
| IVUS guided wiring | 0.165 | 0.017 |
| ADR/RDR | 0.123 | 0.073 |
| AGT | 0.172 | 0.012 |
| Parallel wiring | -0.155 | 0.054 |
| Rotational atherectomy | 0.082 | 0.233 |
| Bridging collaterals usage | -0.107 | 0.121 |
| In-stent CTO | -0.066 | 0.339 |
| Tapered proximal cap | 0.269 | <0.001 |
| Exist of proximal collateral | -0.165 | 0.016 |
| CTO length | -0.219 | 0.001 |
| CTO bend | -0.023 | 0.735 |
| Tapered distal cap | 0.490 | <0.001 |
| Exist of distal collateral | -0.206 | 0.003 |
| Calcification | -0.174 | 0.011 |
| HTN | -0.129 | 0.061 |
| DM | 0.052 | 0.452 |
| HG | 0.014 | 0.834 |
| Smokers | 0.046 | 0.508 |
| LVEF | -0.035 | 0.611 |
| eGFR | -0.039 | 0.575 |
| Prior MI | -0.104 | 0.130 |
| Prior CABG | 0.118 | 0.086 |
| Prior PCI | 0.088 | 0.204 |

CC=collateral connection; CCTA=coronary multi-spiral computed tomography angiography; IVUS=intravascular ultrasound; ADR=anterograde dissection and re-entry; RDR=retrograde dissection and re-entry; AGT=active greeting technique HTN=hypertension; DM=diabetes mellitus; HG=hyperlipidemia; LVEF=left ventricular ejection fraction; eGFR=estimated glomerular filtration rate; MI=myocardial infarction; CABG=coronary artery bypass grafting; PCI= percutaneous coronary intervention.
